# Match running performance in Brazilian professional soccer players: comparisons between successful and unsuccessful teams

**DOI:** 10.1186/s13102-021-00324-x

**Published:** 2021-08-17

**Authors:** Rodrigo Aquino, Luiz Guilherme Gonçalves, Marcos Galgaro, Thiago Santi Maria, Eduardo Rostaiser, Alejandro Pastor, Hadi Nobari, Gabriel Rodrigues Garcia, Maxwell Viana Moraes-Neto, Fábio Yuzo Nakamura

**Affiliations:** 1 LabSport, Department of Sports, Center of Physical Education and Sports, Federal University of Espírito Santo, Vitória, Brazil; 2 School of Physical Education and Sport of Ribeirão Preto, University of São Paulo, Ribeirão Preto, Brazil; 3 Department of Performance Analysis, Botafogo Football Club, Ribeirão Preto, Brazil; 4 Department of Performance Analysis, Juventude Sport Club, Caxias do Sul, Brazil; 5 Catapult Group International Ltd., Melbourne, Australia; 6 Department of Exercise Physiology, Faculty of Sport Sciences, University of Isfahan, 81746-7344 Isfahan, Iran; 7 Sports Scientist, Sepahan Football Club, 81887-78473 Isfahan, Iran; 8 Research Center in Sports Sciences, Health Sciences and Human Development (CIDESD), University Institute of Maia (ISMAI), 4475-690 Maia, Portugal

**Keywords:** Sports science, Match analysis, Contextual factors, Performance, Team sports

## Abstract

**Background:**

This study aimed to compare the match running performance between bottom- and top-ranked teams in professional soccer players over the 2020 season of the Brazilian National 2nd Division League. In addition, this study verified the independent and interactive effects of playing position and contextual factors on running outputs between these teams.

**Methods:**

Forty-eight professional male outfield soccer players participated in this study (top-ranked team, n = 24; bottom-ranked team, n = 24). The distance- and accelerometry-based measures were recorded during 69 matches using a global positioning system (10 Hz) integrated with an accelerometer (400 Hz).

**Results:**

The top-ranked team covered greater total distance [median (interquartile range); 10,330.0 m (1430.0)] and high-acceleration [97.0 m (32.0)] than the bottom-ranked team, in home and away matches [*p* < 0.05, effect size (ES) = small]. The midfielders of the top-ranked team covered higher total distance, high-speed running (> 18 km h^−1^), high acceleration (≥ 3 m s^−2^), high-deceleration (≤ −3 m s^−2^), and performed more sprints [(> 25 km h^−1^) compared to midfielders of the bottom-ranked team (*p* < 0.05, η^2^ = small-moderate]. The matches against top-level opponents required high values of high-acceleration and number of sprints only for the top-ranked team (*p* < 0.05, ES = small). Independent analysis showed that match outcome (loss vs. draw vs. win) was not influenced by running performance for both bottom- and top-ranked teams (*p* > 0.05; η^2^ = small). However, the top-ranked team covered greater total distance, high-acceleration/deceleration than bottom-ranked team in loss matches (*p* < 0.05, η^2^ = small).

**Conclusions:**

These findings should be considered when the coaches and practitioners interpret the match running outputs and when evaluating the effects of training intervention on these performance indicators.

## Introduction

Researchers and practitioners’ interest in the physical performance of soccer players during the match-play using distance-based measures (e.g., distance covered at different speeds) has grown substantially over the last five decades, leading to a myriad of studies and systematic and scientific evidence-based approaches to physical conditioning [1–3]. In addition, global positioning systems (GPS) units are commonly equipped with accelerometers or inertial sensor units (IMUs), which makes it possible to obtain distance- and accelerometry-based data (e.g., acceleration/deceleration) simultaneously [4, 5]. However, there are some issues related to the use and interpretation of measures derived from GPS [6, 7]. For example, some measures (e.g., total distance covered and distance in high-speed running) can be highly dependent on positional role, tactical contexts, players’ physical fitness, competitive standard, and contextual factors, among other aspects [4, 8–10]. On the other hand, the investigation of accelerometry-derived measures in response to the aforementioned independent variables should be better investigated during the match play. In fact, many teams still use semiautomatic camera systems during the matches [11], and it can provide only limited information about competition mechanical load.

The extensive coverage of this research agenda on match running performance has forcibly shaped contemporary opinions with researchers and practitioners frequently emphasizing the position and context and their links with match outcomes [1, 12, 13]. Classic studies reported that players’ fitness levels and competitive physical performance at professional standards were superior to those observed in peers at lower-competitive standards [14, 15], suggesting that the match physical component discriminates the best teams and players from their less competitive counterparts. However, previous studies in professional Leagues in England [16, 17], Italy [18], and Brazil [9] demonstrated that lower distances were covered in higher compared to lower standard divisions and teams’ ranking, with substantial variations across some specific playing positions [19]. In contrast, studies in La Liga [20] and German Bundesliga [21] demonstrated that running performance does not affect the teams’ success as defined by the position on the table, indicating the necessity of further studies in different leagues of the world. In addition, there is a need to improve the current holistic approach to data analysis of top- and the bottom-ranked teams of the same season by evaluating interactions between match running performance and other factors, including the playing positions and contextual variables (e.g., match location, quality of opposition, match outcome). This analysis could provide comprehensive information on the factors that can affect running demands during the match-play in successful and unsuccessful teams.

Therefore, this study aimed to compare the match running performance between bottom-ranked and top-ranked teams over the 2020 season of the Brazilian National 2nd Division League. In addition, this study verified the independent and interactive effects of playing position and contextual factors on running outputs between these teams. We expected that a top-ranked team present greater physical demands than the bottom-ranked counterparts. In addition, we expected that playing position and contextual factors impact the running performance.

## Material and methods

### Study design

This study was conducted under non-experimental conditions in which the research problem was embedded [22]. The players’ running performance was quantified during the matches over the 2020 season of the Brazilian National 2nd Division League. This League was disputed by 20 soccer clubs, of which the top four had access to 1st Division of 2021 and the last four were relegated to 3rd Division of 2021. Originally, the League was scheduled to start on May 2 of 2020, and to end on 28 November of 2020. However, due to the COVID-19 pandemic, its beginning was rescheduled for August 7 of 2020, and the new closure on January 29, 2021.

The present study analyzed the season of one successful team (top-four ranking and access to 1st Division of 2021; n = 31 matches; n = 166 individual observations) and one unsuccessful team (last-four ranking and relegated to 3rd Division of 2021; n = 38 matches; n = 228 individual observations) (Table 1). Five analyses were performed: (1) general results, comparisons of the running outputs between top-and bottom-ranked teams (1st halves vs. 2nd halves vs. whole matches); (2) independent and interactive effects of playing positions (i.e., central defenders, external defenders, midfielders, forwards) and teams’ ranking on running performance; (3) independent and interactive effects of match location (i.e., home, away) and teams’ ranking on running performance; (4) independent and interactive effects of quality of opposition (i.e., bottom-level [16th–20th in the current ranking], intermediate-level [5th–15th in the current ranking], top-level [1st–4th in the current ranking]) and teams’ ranking on running performance; (5) independent and interactive effects of match outcome (i.e., loss, draw, win) and teams’ ranking on running performance.

**Table 1:** Information about the successful and unsuccessful teams analyzed in this study according to the final classification during the 2020 season of the Brazilian National 2nd Division League

Position	Team	Points	W	D	L	Pl	
1	Chapecoense	73	20	13	5	38	Access to the 2021 season of the Brazilian National 1st Division League
2	América-MG	73	20	13	5	38	
3	**Juventude**	61	17	10	11	38	
4	Cuiabá	61	17	10	11	38	
5	CSA	58	16	10	12	38	Maintenance to the 2021 season of the Brazilian National 2nd Division League
6	Sampaio Corrêa	57	17	6	15	38	
7	Ponte Preta	57	16	9	13	38	
8	Operário	57	15	12	11	38	
9	Avaí	55	16	7	15	38	
10	CRB	52	15	7	16	38	
11	Cruzeiro	49	14	13	11	38	
12	Brasil de Pelotas	49	11	16	11	38	
13	Guarani	48	13	9	16	38	
14	EC Vitória	48	11	15	12	38	
15	Confiança	46	12	10	16	38	
16	Náutico	44	10	14	14	38	
17	Figueirense	39	9	12	17	38	Relegation to the 2021 season of the Brazilian National 3rd Division League
18	Paraná	37	9	10	19	38	
19	**Botafogo-SP**	34	8	10	20	38	
20	Oeste	29	7	8	23	38	

W = matches won; D = matches Drawn; L = matches lost; Pl = played matches. Bold means the references teams analyzed in this study. Juventude = successful team; Botafogo-SP = unsuccessful team

### Participants

Forty-eight professional male outfield soccer players participated in this study, including 24 players of top-ranked team (age 28 ± 5 yrs; height 180 ± 5 cm; body mass 78 ± 8 kg; central defenders = 7; external defenders = 5; midfielders = 8; forwards = 5) and 24 players of bottom-ranked team (age 25 ± 5 yrs; height 179 ± 8 cm; body mass 79 ± 9 kg; central defenders = 6; external defenders = 5; midfielders = 7; forwards = 6). Inclusion required participation in ≥ 90 min of play. The study was approved by the local Human Research Ethics Committee (School of Physical Education and Sport of Ribeirão Preto, University of São Paulo; protocol no. 61884716.9.0000.5659).

### Dependent measures

The distance- and accelerometry-based measures were recorded in real-time during the matches using a wearable 10-Hz GPS integrated with a 400-Hz Tri-Axial accelerometer and 10-Hz Tri-Axial magnetometer (Playertek, Catapult Innovations, Australia). The devices were fitted to the upper back of each player using adjustable harnesses and were activated 15 min before the data collection, in accordance with the manufacturer’s instructions to optimize the acquisition of satellite signals. Previous studies analyzed data obtained from this system [23, 24]. The players used the same device throughout the season to avoid inter-unit error [25]. The following metrics were obtained: (1) total distance covered (TD, m); (2) total distance covered under high-speed running (HSR, > 18 km h^−1^, m); (3) number of sprints (> 25 km h^−1^); (4) total distance covered under high-acceleration (Acc, ≥ 3 m s^−2^, m); (5) total distance covered under high-deceleration (Dec, ≤  −3 m s^−2^, m). The speed and accelerometry thresholds used are similar to those reported in previous studies [23, 24, 26].

### Independent measures

Four independent variables were considered for data analysis: (1) playing positions for each player for each match were determined by a Brazilian Soccer Confederation qualified coach and heatmap obtained of the GPS analysis (central defenders [n = 136 individual observations], external defenders [n = 96 individual observations], midfielders [n = 100 individual observations], forwards [n = 61 individual observations]. The tactical formation of the analyzed teams (e.g., 1-4-4-2 “diamond”) and the playing position verified in the heatmap analysis did not allow to divide the midfielders into the two positions usually adopted: central and external; (2) match location (home [n = 205 individual observations], away [n = 188 individual observations]; (3) quality of opposition (bottom-level [n = 71 individual observations]; intermediate-level [n = 250 individual observations]; top-level [n = 72 individual observations]; (4) match outcome (loss [n = 163 individual observations], draw [n = 107 individual observations], win [n = 123 individual observations]).

### Statistical analysis

The Kolmogorov–Smirnov (general results) revealed that match running performance data were not normally distributed for some variables (*p* < 0.05). Thus, to avoid textual confusion, all the data are described by the median (interquartile range). The comparisons of running outputs between bottom- and top-ranked teams (general results) were assessed using the Mann–Whitney test. The ANOVA two-way was used to compare the interactive effects of independent measures on running performance: (1) playing position according to ranking-teams; (2) match location according to ranking-teams; (3) quality of opposition according to team’s ranking; (4) match outcome according to ranking-teams. The significance level was set at *p* < 0.05. Data were analyzed using the SPSS for Windows statistical software package version 22.0 (SPSS Inc., Chicago, IL, USA). Additionally, effect sizes (ES) for non-parametric data (general results) were calculated for pairwise comparisons (ES = z/√n) and classified as negligible (< 0.1), small (0.1–0.29), medium (0.3–0.49), and large (> 0.5) [27]. The ES for parametric data (playing positions, match location, quality of opponents, match outcome) were assessed using partial eta squared (η2), and classified as: > 0.01(small), > 0.06 (moderate), and > 0.15 (large) [28].

## Results

### General

The top-ranked team presented greater TD and high-acceleration during the 1st halves, 2nd halves, and whole matches compared to bottom-ranked team (*p* < 0.001–0.04; ES = small; Table 2).

**Table 2:** Comparisons of match running performance between bottom- vs. top-ranked teams during the 2020 season of the Brazilian National 2nd Division League

Variables	1st Halves		2nd Halves		Whole matches	
	Bottom-ranked Team	Top-ranked Team	Bottom-ranked Team	Top-ranked Team	Bottom-ranked Team	Top-ranked Team
TD (m)	4944.5 (567.5)	5200.0 (770.0)^a^	4918.5 (622.5)	5160.0 (770.0)^a^	9892.0 (1119.5)	10330.0 (1430.0)^a^
HSR (m)	496.0 (266.2)	519.0 (859.0)	473.5 (267.7)	480.0 (322.0)	984.5 (518.5)	1057.0 (660.0)
Number of Sprints	16.0 (9.0)	17.0 (10.0)	15.5 (9.0)	16.0 (9.0)	32.0 (18.0)	34.0 (21.0)
High-acceleration (m)	45.5 (15.0)	48.0 (19.0)^a^	42.0 (13.0)	46.0 (17.0)^a^	87.0 (23.0)	97.0 (32.0)^a^
High-deceleration (m)	52.0 (14.0)	53.0 (22.0)	48.0 (15.0)	51.0 (18.0)	99.0 (25.7)	104.0 (37.0)

The data are described by the median (interquartile range)

TD = Total distance covered; HSR = High-speed running (> 18 km h^−1^); High-acceleration (> 3 m s^2^); High-deceleration (< −3 m s^2^); Number of sprints (> 25 km h^−1^)

^a^Top-ranked Team > Bottom-ranked Team (*p* < 0.05)

### Playing positions

**Fig. 1:**
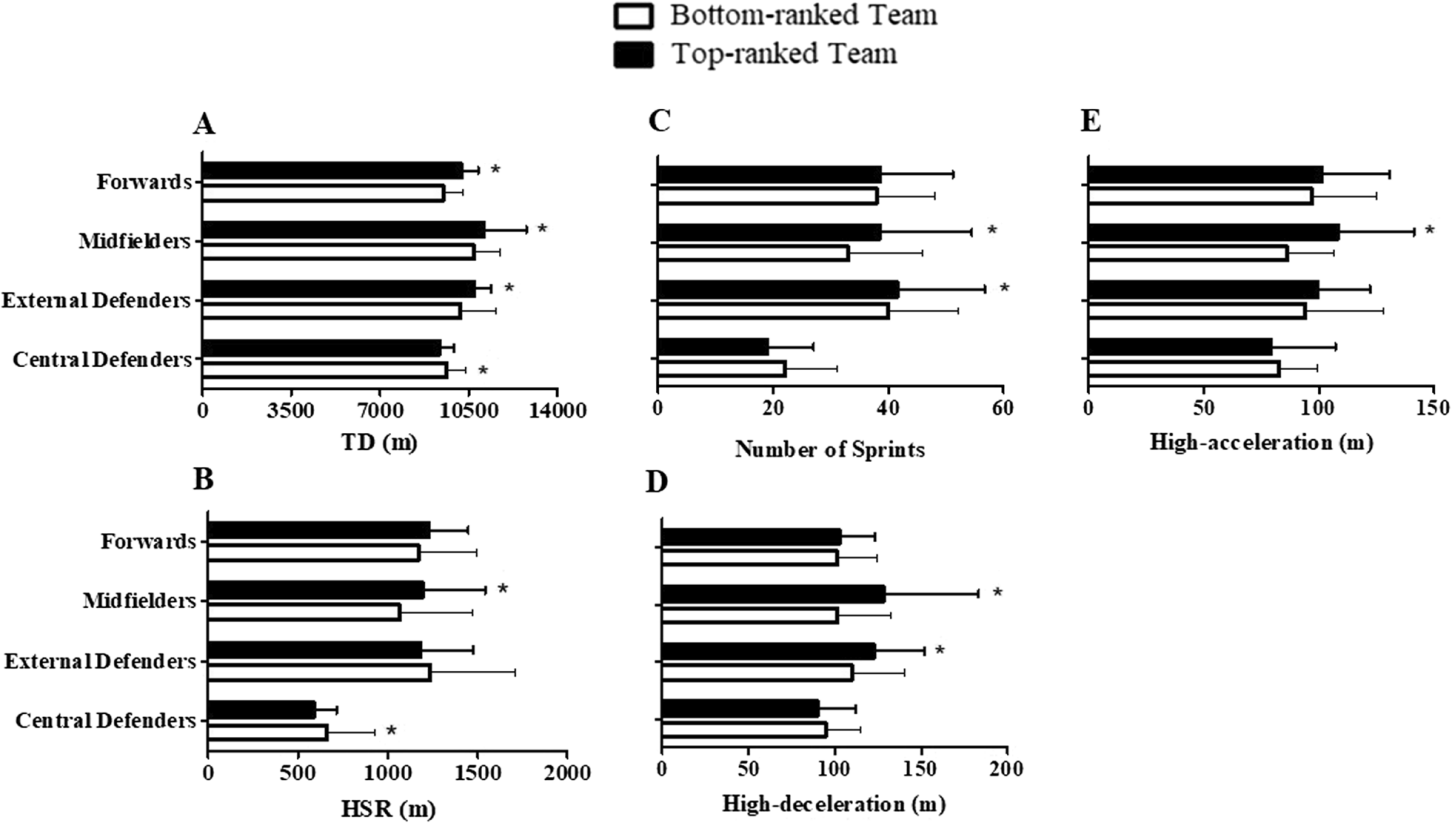
Comparisons of match running performance according to playing positions between bottom- vs. top-ranked teams during the 2020 season of the Brazilian National 2nd Division League. The data are described by the median (interquartile range) of the whole matches. *p < 0.05. TD = Total distance covered; HIR = High-speed running (meters; > 18 km h^−1^); High-acceleration (meters; ≥ 3 m s^−2^); High-deceleration (meters; ≤ −3 m s^−2^); Number of sprints (> 25 km h^−1^)

External defenders (η^2^ = small), midfielders (η^2^ = moderate), and forwards (η^2^ = small) of the top-ranked team covered greater TD than bottom-ranked team (*p* < 0.001–0.004) (Fig. 1A). External defenders of top-ranked team also performed greater number of sprints (η^2^ = small; Fig. 1C) and high-deceleration (*p* η^2^ = small; Fig. 1D) compared to bottom-ranked team (*p* < 0.001–0.03). Midfielders of the top-ranked team showed greater HSR (η^2^ = small; Fig. 1B), number of sprints (η^2^ = small; Fig. 1C), high-acceleration (η^2^ = moderate; Fig. 1E), and high-deceleration (η^2^ = small; Fig. 1D) than bottom-ranked team (*p* < 0.001–0.003). In contrast, central defenders of the bottom-ranked team presented higher TD (η^2^ = small; Fig. 1A) and HSR (η^2^ = small; Fig. 1B) than top-ranked team (*p* = 0.002–0.004).

### Match location

Interactive effects showed that home matches played by top-ranked team resulted in higher TD and high-deceleration compared to home matches played by bottom-ranked team (*p* = 0.01–0.02, η^2^ = small; Table 3). In addition, away matches played by top-ranked team showed greater TD and high-acceleration than away matches played by bottom-ranked team (*p* = 0.001–0.04, η^2^ = small.

**Table 3:** Comparisons of match running performance according to match location between bottom- vs. top-ranked teams during the 2020 season of the Brazilian National 2nd Division League

Variables	Bottom-ranked team		Top-ranked team	
	Home	Away	Home	Away
TD (m)	9916.0 (1257.5)	9829.5 (1123.0)	10440.0 (1310.0)^a^	10290.0 (1567.5)^a^
HSR (m)	1007.5 (502.2)	982.0 (527.2)	1081.0 (715.0)	1001.0 (624.0)
Number of sprints	33.0 (17.0)	31.5 (18.5)	34.0 (20.0)	33.0 (19.7)
High-acceleration (m)	86.0 (24.5)	88.0 (23.0)	95.0 (33.0)	97.5 (29.0)^a^
High-deceleration (m)	98.0 (25.7)	100.0 (25.7)	105.0 (35.0)^a^	103.0 (36.2)

The data are described by the median (interquartile range) of the whole matches

TD = Total distance covered; HSR = High-speed running (> 18 km h^−1^); High-acceleration (> 3 m s^2^); High-deceleration (< −3 m s^2^); Number of sprints (> 25 km h^−1^)

^a^Top-ranked Team > Bottom-ranked Team (*p* < 0.05); *p* < 0.05

### Quality of opposition

Independent analysis showed that top-ranked team covered greater distance in high-acceleration and performed more sprints in matches against top-level opponents compared to bottom-level (*p* = 0.02–0.03, η^2^ = small) (Table 4). Interactive analysis demonstrated that the top-ranked team covered greater TD than the bottom-ranked team in matches against top-level and intermediate-level opponents (*p* < 0.001–0.009; η^2^ = small). Top-ranked team also presented higher values of high-acceleration and high-deceleration than bottom-ranked team in matches against top-level opponents (*p* < 0.001, η^2^ = small).

**Table 4:** Comparisons of match running performance according to quality of opposition between bottom- vs. top-ranked teams during the 2020 season of the Brazilian National 2nd Division League

Variables	Bottom-ranked team			Top-ranked team		
	vs. Bottom-level	vs. Intermediate-level	vs. Top-level	vs. Bottom-level	vs. Intermediate-level	vs. Top-level
TD (m)	10075.0 (979.5)	9941.0 (1190.5)	9643.5 (1025.0)	10120.0 (1300.0)	10340.0 (1567.5)^c^	10,755.0 (1305.0)^c^
HSR (m)	991.0 (549.5)	1004.0 (541.5)	921.5 (480.5)	968.0 (664.5)	1056.0 (637.0)	1201.0 (711.5)
Number of Sprints	33.0 (16.5)	32.0 (18.0)	31.0 (16.5)	29.0 (19.5)	34.0 (20.7)	40.0 (16.5)^b^
High-acceleration (m)	91.0 (29.5)	88.0 (23.5)	83.5 (24.5)	90.0 (37.5)	95.0 (30.0)	107.5 (30.5)^b,c^
High-deceleration (m)	98.0 (26.5)	101.0 (26.0)	95.0 (24.2)	102.0 (41.5)	102.0 (36.5)	117.0 (33.0)^**c**^

The data are described by the median (interquartile range) of the whole matches

TD = Total distance covered; HSR = High-speed running (> 18 km h^−1^); High-acceleration (> 3 m s^2^); High-deceleration (< −3 m s^2^); Number of sprints (> 25 km h^−1^)

^a^vs. Top-level > vs. Intermediate-level (*p* < 0.05)

^b^vs. Top-level > vs. Bottom-level (*p* < 0.05)

^c^ = Top-ranked team > Bottom-ranked team (*p* < 0.05)

### Match outcome

Interactive analysis demonstrated that top-ranked team covered greater TD, high-acceleration, and high-deceleration than bottom-ranked team in loss matches (*p* = 0.004–0.007; η^2^ = small; Table 5).

**Table 5:** Comparisons of match running performance according to match outcome between bottom- vs. top-ranked teams during the 2020 season of the Brazilian National 2nd Division League

Variables	Bottom-ranked team			Top-ranked team		
	Loss	Draw	Win	Loss	Draw	Win
TD (m)	9657.5 (1176.0)	10070.5 (1067.2)	10053.0 (1080.0)	10330.0 (1600.0)^c^	10250.0 (1040.0)	10370.0 (1520.0)
HSR (m)	975.0 (519.5)	987.0 (451.2)	1056.0 (590.2)	1069.0 (640.0)	1068.0 (660.5)	1031.0 (609.0)
Number of Sprints	31.5 (17.0)	33.0 (17.0)	33.5 (19.7)	37.0 (25.0)	35.0 (18.5)	30.0 (19.5)
High-acceleration (m)	85.5 (23.5)	86.5 (19.2)	90.5 (33.2)	100.0 (35.0)^c^	98.0 (29.0)	92.0 (30.0)
High-deceleration (m)	98.5 (24.5)	100.5 (25.7)	100.5 (31.2)	104.0 (40.0)^c^	105.0 (33.5)	100.0 (42.0)

The data are described by the median (interquartile range) of the whole matches

TD = Total distance covered; HSR = High-speed running (> 18 km h^−1^); High-acceleration (> 3 m s^2^); High-deceleration (< −3 m s^2^); Number of sprints (> 25 km h^−1^)

^a^vs. Win > Draw (*p* < 0.05)

^b^Win > vs. Loss (*p* < 0.05)

^c^ = Top-ranked team > Bottom-ranked team (*p* < 0.05)

## Discussion

This study aimed to compare the match running performance between successful (top-ranked) and unsuccessful (bottom-ranked) professional soccer teams. The main findings were: (1) the top-ranked team covered greater TD and high-acceleration than the bottom-ranked team in home and away matches, mainly due to midfielders (moderate effect size). Central defenders presented contrasting results (bottom-ranked team > top-ranked team; small effect size); (2) the matches against top-level opponents required high values of GPS-derived outputs only for the top-ranked team; (3) the match outcome was not influenced by running performance for both bottom- and top-ranked teams. However, the top-ranked team presented greater running demands than the bottom-ranked team in loss matches.

Match running performance was previously investigated in several countries [3]. In general, players covered an average of ~ 9 to 11 km during the matches of different Leagues, including Serie A—Italy [29], Ekstraklasa—Poland [30], League 1—France [31], Eliteserien—Norway [7], La Liga—Spain [32], Premier League—England [33], Croatian League—Croatia [34], or 2nd Division of the Greek League [35]. In our study, both teams presented running outputs similar to the Brazilian elite-level (1st Division: ~ 10 km [36]). Specifically related to HSR, top- and bottom-ranked teams analyzed in this study presented similar values (~ 1000 m above 18.0 km h^−1^) between them and compared to the previous edition (season 2019) of the Brazilian National 2nd Division League [23]. The speed thresholds used to consider HSR in the current study (> 18.0 km h^−1^) hinder direct comparisons between our data and those observed in elite-level domestic Leagues around the world (~ 830 m above 19.1 km h^−1^ or ~ 950 m above 19.8 km h^−1^) [3].

In 2017/18 Serie A League (Italy), the teams within the first four positions showed a lower percentage of running activity, a higher rate of jogging and sprint activities than the teams ranked fifth and below in the same competition [37]. Similar results were found in Chinese Super League [38]. In contrast, previous studies in La Liga (season 2013/14) [20] and German Bundesliga (season 2012/13) [21] reported that both successful and unsuccessful teams presented the same running requirements in total distance covered and total distance or number of activities in HSR (La Liga: meters above 21 and 24 km·h^−1^; German Bundesliga: frequency of running above 18.0 km·h^−1^). Our study identified that top-ranked team covered greater TD and high-acceleration than the bottom-ranked team in home and away matches. These results suggest that high volume of distance covered, and mechanical load were related to success. In general, the contrasting findings between our study (Brazilian National 2nd Division) and previous findings (e.g., La Liga, German Bundesliga) suggest that the influence of the match running performance on a team’s success depends, at least in part, on the disputed League. Specific reasons for this difference can be better investigated in further studies (e.g., comparisons of the physical fitness tests between successful and unsuccessful Brazilian soccer teams in the same competition). In addition, we verified that the magnitude of the differences of the match running performance between top- and bottom-ranked teams were position-dependent.

In English Premier League (season 2003/2004; 2004/2005; 2005/2006), external midfielders of middle- and bottom-ranked teams completed greater HSR (large effect size) than top-ranked teams [19]. In our study, central defenders of the bottom-ranked team presented higher TD and HSR than top-ranked team (small effects sizes). In contrast, we observed that midfielders of the top-ranked team covered greater TD and high acceleration than bottom-ranked (moderate effects sizes). Therefore, despite the previous evidence of small effect size in the comparisons of running performance between top- and bottom-ranked teams (grouped players), moderate-to-large effects were computed when considered playing positions. Our results suggest that some positional rules should be physically prepared to compete in top- (midfielders) and bottom-ranked (central defenders) Brazilian soccer teams. These differences are possibly caused by the more defensive and offensive requirements during the competition in the bottom- and top-ranked teams, respectively.

Research examining contextual factors such as match location, quality of opposition, and match outcome demonstrates these have an impact on the running demands of players [8, 12, 33]. In general, home matches played against top-ranked teams and with a win resulted in greater running outputs [13, 29, 39]. In addition, elite-level players usually performed less HSR when winning than when they were losing [40]. In our study, these contextual factors did not promote independent effects on running performance in both top- and bottom-ranked teams. This findings are in line with previous study during the 2019 season of the Brazilian National 2nd Division League [23]. In addition, the current study observed that independent of the match location, top-ranked team covered greater distances than bottom-ranked team, although with small effect sizes. However, the matches against top-level opponents required high values of high-acceleration and number of sprints only for the top-ranked team, possibly indicating that need to remain among the top-four required more physical effort from the players. These results suggest that players moderate their maximal physical capacity during the match based upon the match characteristics in combination with the opposition [8, 38].

The previous studies in Brazilian National 4th and 3rd Division reported greater match running performance (e.g., TD, HSR) when the teams won in comparison with matches it lost [39, 41]. In the Brazilian National 2nd Division (season 2019), both win and loss matches presented the same running requirements [23], suggesting more stable running outputs in higher-division independent of the match outcome. However, these studies did not consider the team’s final ranking. In our study, the top-ranked team presented greater TD, high acceleration, and high deceleration than the bottom-ranked team in loss matches (small effect size). The descriptive analysis between win, draw, and loss matches within groups showed that the top-ranked team presented more stable running outputs (e.g., TD-loss = 10,330.0 m vs. TD-win = 10,370.0 m) compared to the bottom-ranked team during the competition (e.g., TD-loss = 9567.5 m vs. TD-win = 10,053.0) (see Table 5). We might suppose that the successful team in the league utilizes their physical capacity independent of the match outcome, and when a team is lower in the league their reduce the distances covered and mechanical load when losing the matches. In addition, overall technical-tactical effectiveness probably has a greater impact on match outcome and team’s final League ranking than running performance in elite divisions [1]. For example, the players of the champion team of the 2018 FIFA World Cup (France) and one of the teams with the worst performance (Panama) had a similar match running performance (TD =  ~ 10 km; ~ 10% > 20 km h^−1^). In contrast, French players presented higher levels of interconnectivity between close teammates (greater cooperation) [42].

This study has some limitations that should be recognized. The major limitation was the analyzed data from only successful and one unsuccessful team. The running performance of one team can be affected by some specific factors like style of the play or tactical formation [12]. However, both the analyzed teams presented similar playing styles and tactical formation over the season. Second, the lack of analysis of technical-tactical performance indicators (e.g., notational analysis, collective dynamics, tactical behavior) can be considered other limitation. Third, the design was conducted only in one season. Further studies should investigate the characteristics of successful and unsuccessful teams in multiple seasons and with a holistic data approach (e.g., physical, and technical-tactical).

However, this study advances in some aspects from previous literature about this topic. For example, we considered independent and interactive effects of a myriad of independent measures (i.e., playing positions, match location, quality of opposition, match outcome). Furthermore, to the best of our knowledge, this is the first study that provides information regarding match running performance of successful and unsuccessful teams in Brazilian soccer. These results can aid coaches and practitioners to understand the set of physical variables that better discriminates successful and unsuccessful professional soccer teams, including aspects from the playing position and context that matters.

## Conclusions

This study demonstrated that changes in running performance during match-play are related to several factors, such as the team’s ranking, the playing positions, the match location, the quality of opposition, and the match outcome. Specifically, mainly midfielders of the top-ranked team completed greater running distances than their counterparts of the bottom-ranked team, both in home and away matches. In contrast, central defenders of the bottom-ranked team presented greater running outputs (i.e., TD, HSR) than their counterparts of the top-ranked team. Our results also showed that the quality of opposition influenced only the top-ranked team, which matches against top-level opponents required high values of running performance. Finally, the top-ranked team presented greater TD, high acceleration, and high deceleration than the bottom-ranked team in loss matches. These findings suggest that running performance was an important aspect to discriminate top- and bottom-ranked teams in the Brazilian context.

In addition, strategy and technical-tactical dimensions probably have a greater impact on results and final ranking than running performance. Further studies should investigate these aspects in Brazilian leagues. Therefore, inferences about the cause-effect between match running demands and competitive standard/match outcome should be viewed with caution.

## Data Availability

The datasets generated during and analyzed during the current study are available from the corresponding author on reasonable request.

## Abbreviations

GPS: Global positioning systems
IMUs: Inertial sensor units
TD: Total distance covered
HSR: Total distance covered under high-speed running
Acc: Total distance covered under high acceleration
Dec: Total distance covered under high deceleration
A.U.: Arbitrary units
